# Innate Memory Reprogramming by Gold Nanoparticles Depends on the Microbial Agents That Induce Memory

**DOI:** 10.3389/fimmu.2021.751683

**Published:** 2021-11-04

**Authors:** Benjamin J. Swartzwelter, Sara Michelini, Tobias Frauenlob, Francesco Barbero, Alessandro Verde, Anna Chiara De Luca, Victor Puntes, Albert Duschl, Jutta Horejs-Hoeck, Paola Italiani, Diana Boraschi

**Affiliations:** ^1^ Institute of Biochemistry and Cell Biology (IBBC), National Research Council (CNR), Napoli, Italy; ^2^ Department Biosciences, Paris Lodron University of Salzburg (PLUS), Salzburg, Austria; ^3^ Institut Català de Nanociència i Nanotecnologia (ICN2), Consejo Superior de Investigaciones Científicas (CSIC) and The Barcelona Institute of Science and Technology (BIST), Barcelona, Spain; ^4^ Vall d’Hebron Research Institute (VHIR), Barcelona, Spain; ^5^ Institució Catalana de Recerca I Estudis Avançats (ICREA), Barcelona, Spain; ^6^ Stazione Zoologica Anton Dohrn, Napoli, Italy; ^7^ Shenzhen Institute of Advanced Technology (SIAT), Chinese Academy of Sciences (CAS), Shenzhen, China

**Keywords:** innate immunity, innate memory, nanoparticles, microbial agents, monocytes

## Abstract

Innate immune memory, the ability of innate cells to react in a more protective way to secondary challenges, is induced by exposure to infectious and other exogeous and endogenous agents. Engineered nanoparticles are particulate exogenous agents that, as such, could trigger an inflammatory reaction in monocytes and macrophages and could therefore be also able to induce innate memory. Here, we have evaluated the capacity of engineered gold nanoparticles (AuNPs) to induce a memory response or to modulate the memory responses induced by microbial agents. Microbial agents used were in soluble *vs*. particulate form (MDP and the gram-positive bacteria *Staphylococcus aureus*; β-glucan and the β-glucan-producing fungi *C. albicans*), and as whole microrganisms that were either killed (*S. aureus*, *C. albicans*) or viable (the gram-negative bacteria *Helicobacter pylori*). The memory response was assessed *in vitro*, by exposing human primary monocytes from 2-7 individual donors to microbial agents with or without AuNPs (primary response), then resting them for 6 days to allow return to baseline, and eventually challenging them with LPS (secondary memory response). Primary and memory responses were tested as production of the innate/inflammatory cytokine TNFα and other inflammatory and anti-inflammatory factors. While inactive on the response induced by soluble microbial stimuli (muramyl dipeptide -MDP-, β-glucan), AuNPs partially reduced the primary response induced by whole microorganisms. AuNPs were also unable to directly induce a memory response but could modulate stimulus-induced memory in a circumscribed fashion, limited to some agents and some cytokines. Thus, the MDP-induced tolerance in terms of TNFα production was further exacerbated by co-priming with AuNPs, resulting in a less inflammatory memory response. Conversely, the *H. pylori*-induced tolerance was downregulated by AuNPs only relative to the anti-inflammatory cytokine IL-10, which would lead to an overall more inflammatory memory response. These effects of AuNPs may depend on a differential interaction/association between the reactive particle surfaces and the microbial components and agents, which may lead to a change in the exposure profiles. As a general observation, however, the donor-to-donor variability in memory response profiles and reactivity to AuNPs was substantial, suggesting that innate memory depends on the individual history of exposures.

## Introduction

Immunological memory was long considered a distinctive trait of adaptive immunity, resulting in the capacity to mount a more rapid and more effective specific immune response to infectious challenges (1). It is however evident that organisms that only display innate immunity, the most ancient non-specific defensive system, can develop an immunological memory that allows them to resist better to various environmental pathogens and stressful events (*e.g.*, heat, wounds) (2–4). Higher vertebrates maintain an efficient innate immunity, in parallel to adaptive responses, and it is now evident that priming/exposure to microbial/stressful agents generates “innate memory” in innate immune cells, such as monocytes and macrophages. The innate memory is at least partially non-specific and allows for a more protective reaction to subsequent challenges (2, 3, 5–7).

The first type of innate memory described in mammals is known as “endotoxin tolerance” and results in a less potent secondary response to gram-negative endotoxin or other bacterial challenges, aiming at attaining sufficient protection while avoiding the substantial damage to the host tissues and organs that can be caused by a full innate/inflammatory response, which includes the deadly endotoxin shock (8–11). In other cases, *e.g.*, in the case of exposure to the tuberculosis vaccine BCG or to the fungal β-glucan, the memory response results in a potentiated reaction (“trained immunity”) (6, 12). The innate memory responses, both tolerance and potentiation, are based on epigenetic and metabolic modifications, rather than in a general shift in gene transcription, and they should be understood as a medium-term functional reprogramming aimed at enhanced host defense (lasting several months to years in mammals) (6, 13–16). However, anomalous innate memory has been proposed to contribute to the development of immune/inflammatory diseases, such as autoimmune syndromes and chronic inflammatory diseases (6, 17). Which substances activate innate immune memory, in which direction (protective *vs*. detrimental, tolerance *vs*. potentiation) and how different agents might differentially modulate innate memory is still largely unexplored. Innate memory-inducing substances should be considered both from a safety perspective, in which excessive inflammation or immune suppression can be detrimental, but also for their therapeutic potential, to down-regulate or up-regulate excessive or insufficient innate immunity in different disease conditions.

Several microbial stimuli have been described for their memory inducing capacity. In addition to the aforementioned BCG, endotoxin (lipopolysaccharide -LPS-) and β-glucan, agents such as muramyl dipeptide (MDP) and *Candida albicans* have each demonstrated the capacity to alter the secondary reactivity of monocytes or macrophages (18–20). Recently, several studies have examined whether engineered nanoparticles are also capable of inducing or modulating innate immune memory. While pristine graphene could induce a potentiated status in murine macrophages (21), gold nanoparticles (AuNPs) failed to independently induce a memory response in human monocytes, although they seem able to modulate in different directions the innate memory induced by microbial agents (22–26).

In this context, here we have evaluated the capacity of AuNPs to modulate the innate memory response of human primary monocytes primed with different microbial agents in soluble *vs.* particulate forms (MDP and the gram-positive bacteria Staphylococcus aureus; β-glucan and the β-glucan-producing fungi C. albicans) and with a live microbial agent (the gram-negative bacterium Helicobacter pylori), using a realistic *in vitro* model based on human primary monocytes. The results show that AuNPs are unable per se to induce an inflammatory reaction or to induce innate memory in monocytes, but can partially affect the stimulus-induced cell activation.

## Materials and Methods

### AuNP Synthesis and Characterization

#### AuNP Synthesis

AuNPs were synthesized as previously described by Bastús *et al.* (27). Briefly, 150 mL of sodium citrate 2.2 mM was brought to a boil under reflux, followed by rapid addition of 1 mL of HAuCl_4_ 25 mM. AuNP “seeds” were formed in this manner, and sequential addition of HAuCl_4_ achieved the desired particle size. All reagents were obtained from Sigma-Aldrich^®^ (Merck KGaA, St. Louis, MO, USA).

#### AuNP Characterization

##### Transmission Electron Microscopy and Scanning Electron Microscopy

NP characterization images were obtained by STEM (scanning transmission electron microscopy) using a FEI Magellan XHR microscope (FEI, Hillsboro, OR, USA) in transmission mode with an acceleration of 20 kV, as previously described (22). AuNP samples were stabilized with polyvinylpyrrolidone (55 kDa) (28) and drop cast onto a carbon-coated TEM grid. After drying, samples were imaged and particle size was assessed using an ImageJ macro. Scanning Electron Microscopy (SEM) was conducted on a JEOL 6700F scanning electron microscope (JEOL, Peabody, MA, USA) as described previously (29).

##### UV-vis Spectroscopy

To assess particle stability and uniformity of size, UV-vis spectra of the AuNP suspensions were obtained using a Shimadzu UV-2400 spectrophotometer (SSI, Kyoto, Japan) with a range of 300-700 nm. Samples were measured at room temperature, and milliQ water was used as a reference.

##### Dynamic Light Scattering

Particle ζ-potential and hydrodynamic diameter were determined by laser doppler velocimetry and dynamic light scattering (DLS), respectively, using a Malvern Zetasizer Nano ZS instrument (Malvern Panalytical Ltd., Malvern, UK) with a light source wavelength of 632.8 nm and a fixed scattering angle of 173° (at 25°C).

##### Atomic Force Microscopy

AFM measurements were performed with XE-70 microscope (Park Systems, Suwon, South Korea). The instrument is equipped with two flexure scanners (XY plane and Z) both for probe tips and samples. Scans were performed on an area up to 15x15 µm^2^ with a topographic resolution below 1 nm (30). AFM images were acquired after deposition of AuNPs (10 µL at 1022 µg/mL) on a quartz slide by drop-casting.

##### Evaluation of Endotoxin Contamination

The presence of endotoxin contamination in NP samples was assessed with the Limulus Amoebocyte Lysate assay (LAL). The chromogenic Pyrochrome LAL assay (Associates of Cape Cod, Inc.; East Falmouth, MA, USA) was conducted at NP concentrations determined be to below the threshold for optical interference, following a protocol optimized for NPs (31), and sample absorbance was assessed using a Cytation 3 imaging reader (BioTek, Winooski, VT, USA). Endotoxin levels were expressed as endotoxin units per milligram of AuNPs (EU/mg).

### Human Monocyte Isolation

Primary human monocytes were isolated from buffy coats of 20 healthy anonymous donors (provided by the blood bank of Salzburg, Austria, following overnight refrigeration), with cells from 4-8 buffy coats used for each primary stimulus. The study was conducted in accordance with the Declaration of Helsinki, and under Austrian national guidelines. According to Austrian regulations, no informed consent is required if blood cells derived from anonymous healthy donors, discarded after plasmapheresis (buffy coats) are used, therefore no additional approval by the national ethics committee was necessary. Peripheral blood mononuclear cells were obtained by Ficoll-Paque gradient density separation (GE Healthcare, Bio-Sciences AB, Uppsala, Sweden). Monocytes were further isolated by CD14^+^ magnetic microbead separation (Miltenyi Biotec, Bergisch Gladbach, Germany) following the manufacturer’s instructions. The resulting cell suspension was monitored for purity by differential counting on Wright-Giemsa-stained cytosmears (Diff-Quik; Medion Diagnostics, Düdingen, Switzerland) examined by optical microscopy. Cell viability was assessed by trypan blue dye exclusion. Only cell isolations with at least 95% purity and 95% viability were used.

### Human Monocyte Primary Stimulation and Innate Memory Response

#### AuNP Biocorona Formation

Before addition into cell culture, AuNPs were pre-incubated in 50% inactivated human AB serum (Sigma-Aldrich) at 37°C for 1 h, in order to obtain the formation of a bio-corona of serum proteins and other components on the particle surface thereby ensuring particle stability in culture. However, being this a soft corona, it still allowed for interaction of the reactive particle surface with microbial agents and cells in culture (32, 33). The serum-AuNP mixture was added directly to culture wells (34), adjusting particle and serum concentration to the desired values.

#### Monocyte Primary Innate Response

Freshly isolated monocytes were suspended in culture medium (RPMI-1640 + Glutamax-I; GIBCO by Life Technologies, Paisley, UK) supplemented with 100 U/mL penicillin/streptomycin (Sigma-Aldrich). Cells (1x10^5^/well) were added to 96 well flat bottom plates (Corning^®^ Costar^®^; Corning Inc. Life Sciences, Oneonta, NY, USA). Cells were exposed to β-glucan (extracted from *C. albicans*; 2 µg/mL; a generous gift from Charles Dinarello, University of Colorado, Denver CO, USA), MDP (10 µg/mL; InvivoGen, San Diego, CA, USA), heat-killed *S. aureus* (ratio with monocytes 1:1; strain ATCC 6538, InvivoGen), heat-killed *Candida albicans* (ratio 0.1:1; strain ATCC 10231, InvivoGen), or live *H. pylori* (at MOI 0.2, 1, 5; WT strain p12, cultured in-house as described previously) (35). *H. pylori* CFUs were determined by spectrophotometric measurement of bacterial culture turbidity (OD_600_; BioPhotometer plus, Eppendorf, Hamburg, Germany), following an in-house CFU calibration curve.

Cell stimulation was performed in the presence or absence of 20 µg/mL AuNPs. The final serum concentration of each well was adjusted to 5%. The primary monocyte response to stimuli was assessed as cytokine analysis in the 24 h supernatants. For stimulation with *H. pylori*, antibiotics were absent during the primary stimulation to ensure bacterial integrity during the primary activation/memory induction phase. Antibiotics were added into the culture medium for both the resting and challenge phases, to avoid unwanted activation by residual bacteria and to maintain the same culture conditions as for other stimuli.

#### Monocyte Memory Innate Response

After the primary response and supernatant collection, cells were rested in fresh culture medium for 6 days, with medium changes on days 4 and 6. A resting period of 6 days was sufficient for the complete extinction of monocyte activation induced by the different stimuli, based on the production of inflammation-related factors. That monocytes were no longer activated was assessed by measuring cytokine production in the 6-day supernatant (representing the cytokine production from day 4 to 6; data not shown) and by challenging the primed cells with culture medium alone (see first column on the left “challenged by medium” in all the figures reporting innate memory results). At day 7, cells were exposed to fresh culture medium alone of containing 5 ng/mL of LPS (from *Escherichia coli* O55:B5; Sigma-Aldrich). Supernatants were collected after 24 h for cytokine analysis.

### Cytokine Analysis

Production of TNFα and IL-1Ra was measured in the culture supernatants by ELISA (R&D Systems, Inc., Minneapolis, MN, USA). All other cytokines and chemokines were measured using a ProcartaPlex multiplex assay (Thermo Fisher Scientific, Waltham, MA, USA). The lower detection limits for the assays used was: TNFα, 15.6 pg/mL; IL-6, 9.4 pg/mL; IL-1Ra, 93.8 pg/mL; IL-10, 7.1 pg/mL; MCP-1, 15.0 pg/mL; IL-1α, 9.5 pg/mL; MIP-1α, 8.9 pg/mL; MIP-1β, 110.1 pg/mL; GROα, 3.0 pg/mL; IP-10, 23.4 pg/mL; IL-8, 31.2 pg/mL. Two ELISA replicates were run for each sample, and each experimental condition was tested with duplicate samples.

### Statistical Analysis

Cytokine levels are reported as ng/10^6^ plated monocytes. Graphical presentations and statistical analysis were obtained using Graphpad Prism 9 (GraphPad Inc., La Jolla, CA, USA). Data are shown as averages of biological triplicates or as averages of technical replicates of biological duplicates. Statistical analysis was conducted using one-way ANOVA with the Fisher’s LSD *post hoc* test for multiple comparisons. The Shapiro-Wilk normality test was conducted on each data set prior to ANOVA, to ensure normal distributions.

## Results and Discussion

### AuNP Characterization

**Figure 1:**
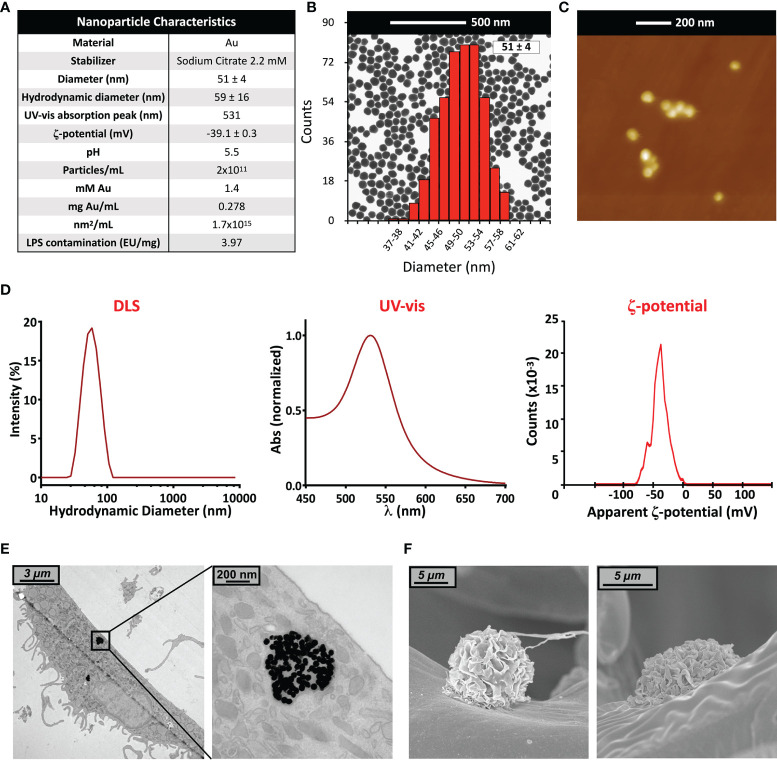
Gold nanoparticle characterization. **(A)** Summary of characteristics of the AuNP batch used in this study; **(B)** TEM image and size distribution (calculated *via* ImageJ); **(C)** Atomic Force Microscopy image; **(D)** UV-vis spectrum, hydrodynamic size distribution calculated by DLS, and ζ-potential; **(E)** TEM and **(F)** SEM images of human primary monocytes, pre-exposed to AuNP for 24 h and then cultured for 6 additional days.

In this study, we used gold nanoparticles (AuNPs in 2.2 mM sodium citrate) of an average diameter of 51 ± 4 nm (**Figures 1A–E**). Particle size and uniformity was confirmed by UV-vis (a single peak found at 531 nm), while DLS revealed a hydrodynamic size of 59 ± 16 nm, and a ζ-potential of about -39 ± 3 mV. The stock concentration following synthesis was 278 µg/mL (corresponding to a particle concentration of 2x10^11^ NPs/mL, to 1.4 mM Au, and to a surface area of 1.7x10^3^ mm^2^/mL), with an endotoxin contamination (determined by LAL assay) of 3.97 EU/mg (**Figure 1A**). Endotoxin may activate monocytes at concentrations above 0.1 EU/mL; our preparation thus allowed for a NP working concentration in culture of 20 µg AuNPs/mL, containing 0.079 EU/mL of endotoxin, which is below the endotoxin activation threshold (36, 37). Prior to addition into culture, AuNP were incubated in 50% AB serum, to better mimic the physiological conditions of NP interaction with human immune cells (38). Formation of a serum-dependent biocorona on the NP surface avoided particle aggregation in culture medium (32, 39). The presence of AuNPs within cells was assessed by TEM 6 days after monocyte exposure to NPs for 24 h (**Figure 1E**). Particles could be observed within endosomes, but not free in the cytosol or within nuclei. This is in agreement with the notion that particles are endocytosed and kept within vesicles for eventual degradation, implying a mechanism of silent, non-inflammatory elimination of foreign/anomalous materials. The absence of AuNPs in the cytoplasm suggests that the particles are unable to destabilize the vesicle membrane and, consequently, to induce the activation of cytoplasmic inflammasomes by released lysosomal enzymes and mitochondrial ROS. Importantly, despite the lack of inflammatory activation, the mechanism of silent elimination was reported as able to “prime” macrophages and induce a protective innate memory *vs*. subsequent challenges (40). At this timepoint, no appreciable differences were noted in intracellular NP number, size and distribution across all experimental conditions (not shown), and no morphological alterations in monocytes were observed (**Figure 1F**).

### Effect of AuNPs on the Primary Innate Response Induced by Soluble *vs.* Particulate Microbial Stimuli

We aimed to determine whether the presence of AuNPs might interfere with the induction of innate immune memory by microbial stimuli. In particular, we wanted to examine possible differential effects on innate memory induced by particulate stimuli (whole microorganisms) or by microbial molecules. Freshly isolated human blood monocytes were exposed for 24 h *in vitro* to culture medium alone or containing one of four microbial agents: the bacterial surface molecule muramyl dipeptide (MDP, 10 µg/mL); heat-killed gram-positive *Staphylococcus aureus* (*S. aureus*, ratio 1:1 with monocyte); the fungal polysaccharide β-glucan (2 µg/mL) and heat-killed *Candida albicans (C. albicans*, at a ratio 0.1:1 with monocytes). Concentrations were selected based on preliminary experiments and literature data as able to induce a significant but suboptimal innate immune activation (19, 41, 42; data not shown).

The direct, primary response of monocytes to microbial stimuli was evaluated in the absence or in the presence of AuNPs (20 µg/mL), and assessed in the 24-h supernatant as production of innate/inflammatory cytokines and chemokines. The size of AuNPs was chosen based upon preliminary data using AuNPs of different sizes, which suggested that 50 nm AuNPs were the best for observing effects on innate memory (23). The concentration was selected as the highest non-toxic and endotoxin-free concentration (data not shown).

**Figure 2:**
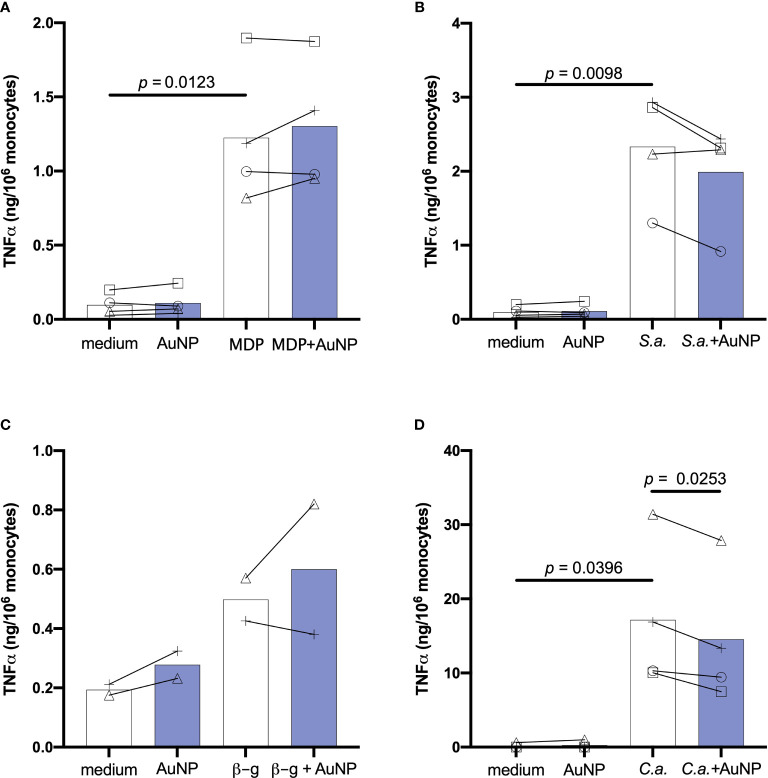
Effect of AuNPs on the primary response of monocytes exposed to soluble or particulate microbial stimuli. CD14^+^ monocytes were exposed to MDP (10 µg/mL) **(A)**, *S. aureus* (ratio 1:1; *S.a.*) **(B)**, β-glucan (2 µg/mL; β-g) **(C)** or **(*C*)** *albicans* (ratio 0.1:1; *C.a.*) **(D)** for 24 h in the presence or absence of AuNPs (20 µg/mL, indicated by blue bars). The inflammatory response is reported in terms of production of TNFα (ng/10^6^ monocytes). Values from individual donors are depicted concurrent with mean cytokine production. Relevant *p* values are indicated when < 0.05. n = 4 **(A, B, D)**, n = 2 **(C)**.

**Figure 2** shows the primary response of monocytes in terms of production of the inflammatory cytokine TNFα, measured in monocytes from 2-4 individual donors. As expected, cells exposed to culture medium alone or containing the endotoxin-free AuNPs did not produce appreciable levels of TNFα (<0.3 ng/10^6^ monocytes). Stimulation of monocytes for 24 h with bacterial MDP or killed gram-positive *S. aureus* resulted in a substantial production of TNFα, which was not overall significantly impacted by the presence of AuNPs, although a decrease in the response to *S. aureus* was evident for cells of 3 out of 4 donors (**Figures 2A, B**). Cells were also stimulated with *C. albicans*-derived β-glucan and with the whole killed *C. albicans* organisms. Both fungal agents also induced TNFα production, although this increase did not attain statistical significance for β-glucan (only two subjects could be tested; **Figure 2C**). Co-exposure of monocytes to *C. albicans* and AuNPs caused a significant suppression of TNFα production in cells from all donors (**Figure 2D**). To investigate whether the AuNP effect observed for *C. albicans*-stimulated TNFα production was common to other *C. albicans*-induced factors, we examined the production of the inflammatory cytokine IL-6, the anti-inflammatory factor IL-1Ra and the chemokine MCP-1/CCL2, and found that AuNPs did not affect the stimulus-induced production of any of them (**Supplementary Figure 1**).

These results confirm previous observations that AuNPs do not have a substantial impact on the innate/inflammatory response of human monocytes to microbial stimuli, but that a partial reduction of the response to whole microorganisms can be observed in the majority of donors. Notably, individual effects can be observed on cells from many donors, these effects being variable (increase or decrease of the response) depending on the donor and irrespective of the inflammatory agent. Only in the case of *C. albicans* was a similar decrease observed in all donors, thus reaching statistical significance (**Figure 2D**).

### Effect of AuNPs on the Memory Innate Response Induced by Soluble *vs.* Particulate Microbial Stimuli

**Figure 3:**
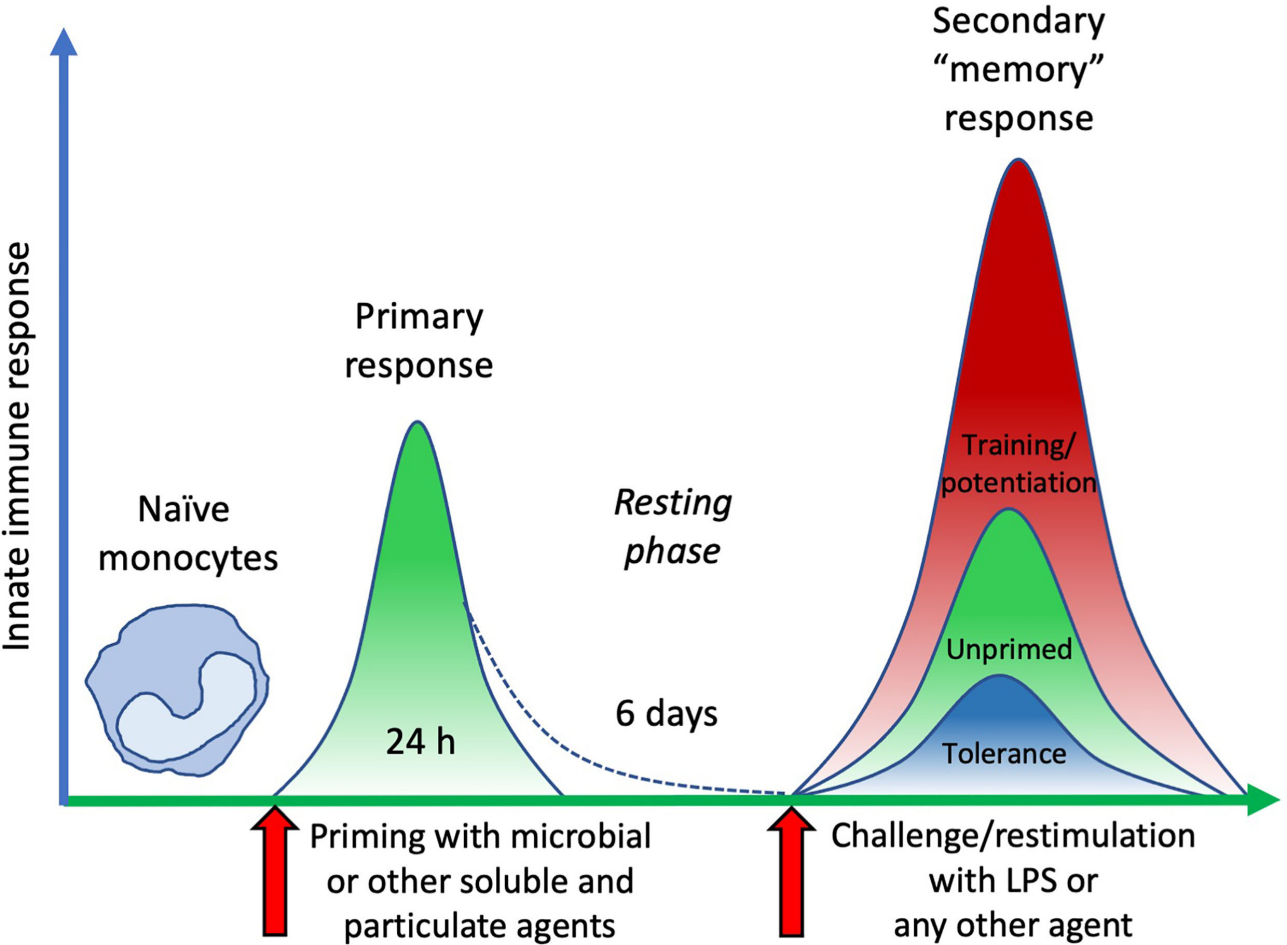
The time course of an *in vitro* model of innate immune memory. Fresh naïve monocytes are activated by exposure in culture to different stimuli for 24 h (primary response). After elimination of stimuli, cell activation subsides with time (in our *in vitro* model 6 days are sufficient), during which period cells return to a resting status. Upon restimulation, cells that were not previously exposed to activating agents (unprimed) develop a secondary response of a given intensity. Conversely, cells that were previously primed and activated can react to restimulation with a secondary “memory” response, either more powerful (training/potentiation) or reduced (tolerance), compared to unprimed cells.

**Figure 4:**
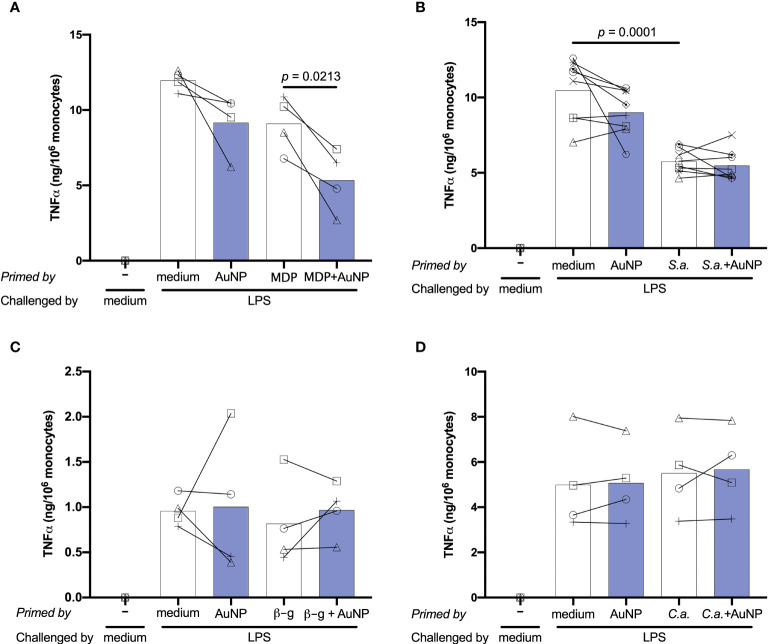
Effect of AuNPs on the memory response of monocytes primed by soluble or particulate microbial stimuli. CD14^+^ monocytes were primed with MDP **(A)**, *S. aureus* **(B)**, β-glucan **(C)** or *C. albicans* **(D)** for 24 h in the presence or absence of AuNPs (20 µg/mL, indicated by blue bars), then washed and rested for 6 days. Cells were then challenged with LPS (5 ng/mL) for 24 h, and supernatants collected for cytokine measurement. The inflammatory response is reported in terms of production of TNFα (ng/10^6^ monocytes), and individual donor values are depicted concurrent with mean cytokine production. *p* values are indicated when < 0.05 for the comparisons between unprimed and primed groups and priming with and without NPs, n = 4 **(A, C, D)**, n = 8 **(B)**.

Following primary activation, cells were rested for 6 days. This allows monocytes sufficient time to return to quiescence, prior to restimulation. Cell number and morphology following resting appeared consistent (by visual inspection) across wells from different primary conditions. After resting, cells were challenged with either medium alone or 5 ng/mL of LPS, in order to observe whether the previous exposure to inflammatory agents resulted in development of an innate immune memory (an increased or decreased response compared to control unprimed cells). The *in vitro* model adopted for assessing memory induction is depicted in **Figure 3**. Challenge with medium alone resulted in TNFα levels below the detection limit of the assay for all primed cells, indicating that cells had returned to baseline TNFα production (**Figure 4**; medium-challenged cells are grouped into one bar that includes every priming condition tested). Restimulation with LPS induced significant production of TNFα, which was comparable between medium- and AuNP-primed cells, suggesting that pre-exposure to AuNPs was unable to induce a consistent memory response. It should be however noted that, while the average production is not statistically different between control and AuNP-primed monocytes, at the individual level there are cases in which AuNP-primed cells respond to challenge with an increased TNFα production, others in which there is a decrease, and others in which there is no change (see for instance the four donors in **Figure 4C**). This again underlines the need for an individual profiling of innate and memory responses, in order to predict reactivity to future challenges. Such profiling should include the production of a number of inflammatory and anti-inflammatory factors in response to different microbial challenges, in order to assess the overall balance between inflammation and anti-inflammation (26). The memory response of MDP-primed cells was of tolerance type, relative to TNFα production, with a decreased production of the inflammatory cytokine compared to medium-primed cells. Also in this case, the global difference was not statistically significant (p=0.0956), due to the interindividual variability (with monocytes from 1 out of 4 donors showing no change). Cells primed with MDP + AuNPs showed a significant tolerance at restimulation, compared to cells primed with either stimulus alone, confirming the tendency to tolerance observed with single priming agents (**Figure 4A**). Restimulation of cells primed with *S. aureus* (which contains MDP as part of its surface structure) demonstrated that, similar to MDP, *S. aureus* is a potent inducer of innate memory in the direction of tolerance (**Figure 4B**). Unlike the memory effect upon MDP priming, AuNPs had no effect on *S. aureus*-driven tolerance. The difference in the effect of AuNPs on memory induced by MDP *vs*. the entire *S. aureus* bacteria might be ascribed to the different mechanisms of primary cell activation (which then initiate the epigenetic and metabolic reprogramming responsible for the establishment of memory), MDP mainly acting through NOD2 in the cytoplasm after receptor-independent endocytosis/transport through membrane channels (43, 44), while the whole bacteria principally interact with the plasma membrane through lipoteichoic acid activation of TLR2, thereby initiating an MyD88-dependent signaling pathway (45, 46). The metabolic cost and pathway involvement of bacterial phagocytosis compared to uptake of soluble factors most likely also contribute to the different memory profiles generated by *S. aureus* and MDP, although this remains unstudied to date (47). Thus, the presence of AuNPs, which are endocytosed, may have interfered with the MDP-dependent mechanism of memory generation, while unable to affect the memory mechanisms initiated extracellularly by *S. aureus*.

Upon challenge, cells primed with the fungal agents β-glucan and *C. albicans* did not demonstrate an innate memory response, as their TNFα production did not differ from that medium-primed (**Figures 4C, D**). In both cases, the presence of AuNPs at priming did not have any effect on the secondary response at challenge. To make sure that the lack of memory induction was not restricted to the production of a single inflammatory factor, in the case of *C. albicans* priming we also assessed the production of another inflammatory cytokine (IL-6), two anti-inflammatory factors (IL-10 and IL-1Ra) and six chemokines (three CC chemokines: MCP-1/CCL2, MIP-1α/CCL3 and MIP-1β/CCL4; and three CXC chemokines: GROα/CXCL1, IP-10/CXCL10 and IL-8/CXCL8) (**Supplementary Figure 2**). Two of these factors (IL-1Ra and CCL2/MCP-1) were spontaneously produced at high levels, and their levels were not increased in response to the LPS challenge. Similar to TNFα, priming with *C. albicans* did not induce memory (either potentiation or decrease of the secondary response) in terms of production of any of these factors. Likewise, the presence of AuNPs at priming did not have any significant effect (**Supplementary Figure 2**).

In all cases, again it should be noted that the interindividual variability is high and that, while the average values are not statistically different, the individual effects can be substantial both as decrease and increase of the memory response in the presence of AuNPs.

### Effect of AuNPs on Primary and Memory Innate Responses Induced by Live Bacteria

**Figure 5:**
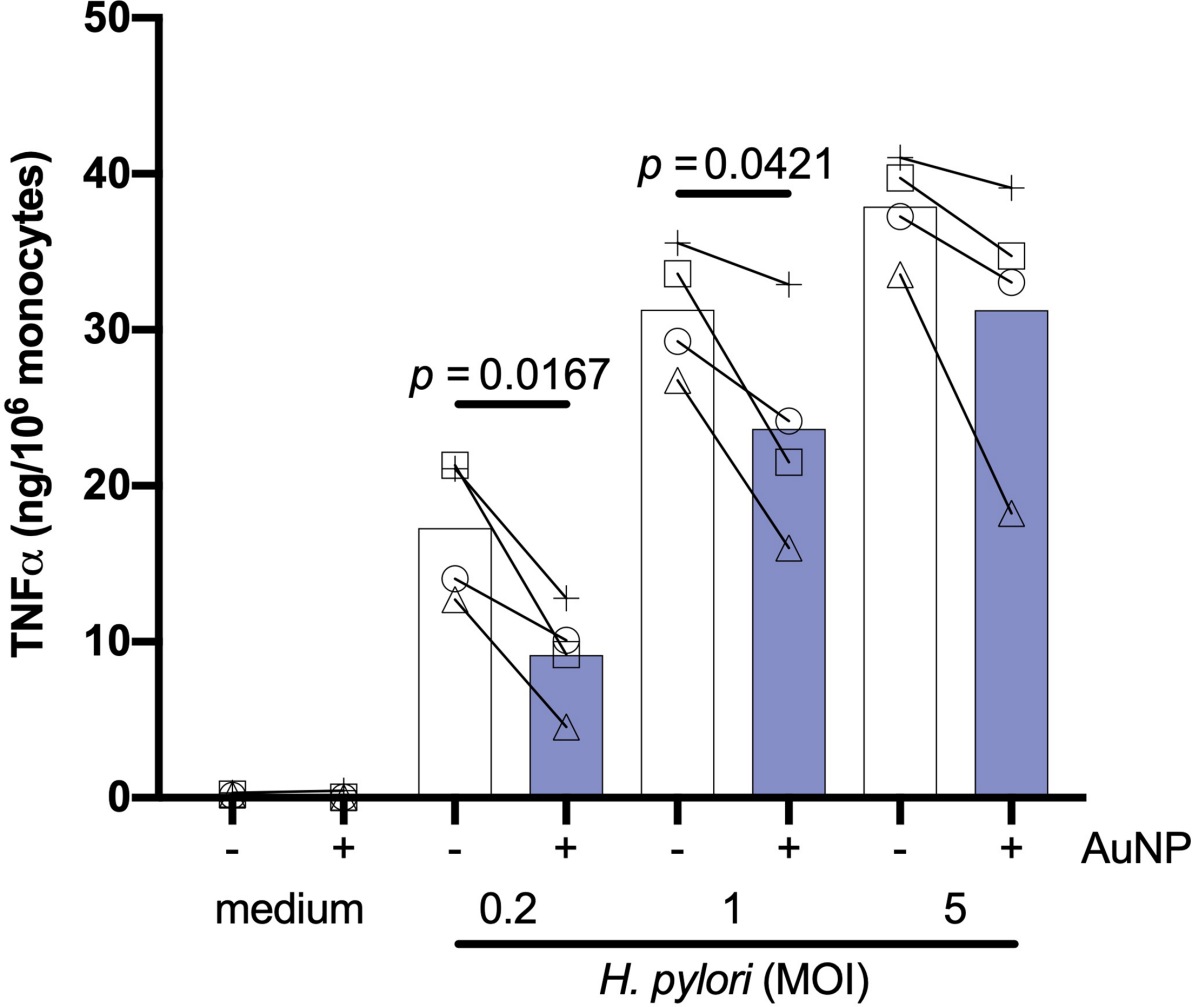
Effect of AuNPs on the primary response of monocytes to live *H. pylori*. CD14^+^ monocytes were stimulated with medium alone or containing live *H. pylori* (MOI: 0.2, 1, 5) for 24 h in the presence or absence of AuNPs (20 µg/mL, indicated by blue bars). The inflammatory response is reported in terms of production of TNFα (ng/10^6^ monocytes). Values from individual donors are depicted concurrent with mean cytokine production. *p* values are indicated when < 0.05 for the comparison between stimulation with or without NPs, n = 4.

Previous data suggest that the impact of AuNPs on innate memory induced by bacteria may vary depending on whether bacteria (BCG in this specific case) are viable or not (23). We have therefore also tested the effect of AuNPs on responses induced by a live microorganism, the gram-negative *H. pylori*, so as to compare such effect with those induced by killed microorganisms (*S. aureus* and *C. albicans*). Monocytes were primed *in vitro* for 24 h by live *H. pylori* at three concentrations (at MOI 0.2, 1 and 5) in the absence or presence of AuNPs. Primary stimulation with *H. pylori* revealed a potent dose-dependent induction of TNFα production (**Figure 5**), which was significantly suppressed by the presence of AuNPs, with the most robust suppression present at the lowest *H. pylori* dose (MOI 0.2). Thus, similar to what was observed with live BCG (23) and, to a lower extent, with killed whole bacteria (**Figures 2B, D**), AuNPs are capable of interfering with the primary innate response induced by live *H. pylori*.

**Figure 6:**
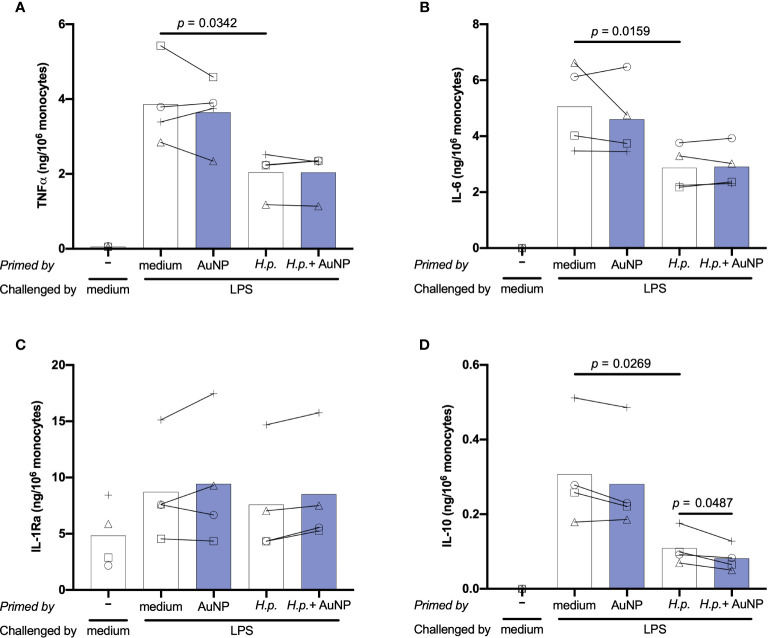
Effect of AuNPs on the memory response of monocytes primed by live (*H*) *pylori*. CD14^+^ monocytes were exposed to medium or *H. pylori* (at MOI 0.2) for 24 h in the presence or absence of AuNPs (20 µg/mL, indicated by blue bars), then washed and rested for 6 days. After resting, cells were challenged with LPS (5 ng/mL) for 24 h, supernatants were collected and examined for the production of TNFα **(A)**, IL-6 **(B)**, IL-1Ra **(C)** and IL-10 **(D)**. Values from individual donors are depicted concurrent with mean cytokine production. *p* values are indicated when < 0.05 for the comparisons between unprimed and primed groups and priming with and without NPs, n = 4. .

Induction of innate immune memory by *H. pylori* was assessed using the same *in vitro* model described previously for soluble and particulate microbial stimuli. Following 6 days of resting, control and primed cells were challenged with 5 ng/ml of LPS for 24 h. To better assess the memory induction by *H. pylori* and the AuNP impact, in addition to TNFα we have examined several other important inflammation-related cytokines and chemokines. Results in **Figure 6** show the memory response of *H. pylori*-primed cells in terms of production of two key inflammatory factors, TNFα and IL-6, and of two anti-inflammatory cytokines, IL-1Ra and IL-10. Additional factors are reported in **Supplementary Figure 2**. Only the results at *H. pylori* MOI 0.2 are shown, since no substantial differences were observed at higher concentrations. Upon challenge with LPS, cells primed with medium exhibited elevated production of all cytokines and chemokines measured, except IL-1Ra and MCP-1, whose baseline levels were already high (**Figure 6** and **Supplementary Figure 3**). Overall, the secondary response of cells primed with AuNPs was not significantly different from that of medium-primed cells, although again different behaviors were evident between donors. The memory response of *H. pylori*-primed cells revealed a potent induction of an innate immune tolerance in terms of TNFα, IL-6, IL-10 and the CXC chemokine IP-10, though not for IL-1α, IL-1Ra and in all the other chemokines tested (**Figure 6** and **Supplementary Figure 3**). The presence of AuNPs during priming with *H. pylori* did not alter the *H. pylori*-induced memory effect on any of the cytokines and chemokines tested, with the exception of the anti-inflammatory cytokine IL 10. In this case, the *H. pylori*-induced tolerance was significantly enhanced by AuNPs (**Figure 6D**), an effect evident at all *H. pylori* priming concentrations (data not shown).

Thus, monocytes exposed to live *H. pylori* can mount a potent inflammatory response that primes cells towards a generally less potent secondary memory response, in terms of production of two inflammatory factors (TNFα and IL-6), an anti-inflammatory cytokine (IL-10) and the chemokine IP-10. That *H. pylori* priming may affect the production of four different cytokines suggests different levels of epigenetic/metabolic reprogramming, likely dependent on the multiplicity of its cell activation modes. In fact, *H. pylori* can interact with TLR2 on the cell membrane, possibly through Hsp60 (48–50), while its LPS has very limited inflammatory activity and does not trigger a significant TLR4-mediated inflammation (51–53). Of note, the *cag* pathogenicity island (*cag*PAI), which mediates *H. pylori* pathogenesis in gastric epithelial cells, is less important in macrophages and dendritic cells (50, 54). *H. pylori* can induce inflammatory activation of innate cells (53, 55–57), possibly through the non-enzymatic interaction of secreted urease with receptors/acceptors on the cell membrane (55, 56), promote the macrophage M1 inflammatory phenotype through NOD1 (58), and survive for at least 24 hours within phagolysosomes after ingestion thereby inducing potent ROS production (59–61). By inducing phosphorylation of the NFκB p65 subunit at Ser-537, also the integrin-like kinase (ILK) promotes *H. plyori*-induced TNFα production (62). All these mechanisms of inflammatory activation will likely induce a multitude of different metabolic and epigenetic changes resulting in a complex innate memory profile. The tolerance memory response observed for TNFα and IL-6, two cytokines mainly dependent on the activation of the NFκB pathway, suggests that the TLR2-dependent priming may be principally involved. Conversely, the effects on IL-10 and IP-10, two factors that largely depend on interferon activation, are more likely mediated by other mechanisms, including the NOD1 pathway through the TRAF3-dependent induction of IRF3 and 7 and the production of type I IFN (63–70), which in turn activates the JAK/STAT signaling pathway (71). In this perspective, the capacity of AuNPs to interfere with *H. pylori*-induced memory, which is evident only in the case of IL-10, suggests that AuNPs may increase the *H. pylori* effect through production of type I IFN.

## Conclusions

The aim of this study was to assess whether engineered AuNPs, a nanomaterial with wide applications in many fields including medicine and generally considered safe, are able to modulate the innate immune/inflammatory responses of human subjects. This would contribute on one side to the implementation of a more thorough safety evaluation of AuNPs and, on the other hand, it could open the way to a targeted use of this nanomaterial for the therapeutic modulation of innate immunity/inflammation in several immune-related and inflammatory diseases. In particular, this study has addressed innate memory, *i.e.*, the ability of monocytes/macrophages to activate a more protective reaction to a challenge when previously exposed to the same or a different infectious agent (6, 7, 9–17). Following previous studies showing that endotoxin-free Au and other NPs are essentially unable to induce innate/inflammatory responses and innate memory *per se*, but could at least in part modulate the memory induced by microbial agents (21–26, 72), here we have examined if the nature of the memory-inducing microbial agents could determine the capacity of AuNPs to interfere with the development of innate memory. To study innate memory, we have taken advantage of a realistic *in vitro* model, based on human primary monocytes exposed to microbial agents and to AuNPs coated with human serum. Based on our preliminary findings, we can draw the following conclusions and formulate the following hypotheses:

1. AuNPs generally decrease the inflammatory activation of monocytes induced by whole microorganisms, both killed (*C. albicans*, *S. aureus*) and viable (*H. pylori* in this study, BCG in ref. 23). Conversely, monocyte activation induced by microbial molecules (β-glucan and MDP in this study, LPS in refs. 22, 23, 25, 26) is not consistently affected by co-exposure to AuNPs. This may be ascribed to a possible interference of AuNPs (which are readily and abundantly taken up by monocytes and stored in endosomal vesicles) with the intracellular trafficking of phagocytosed microorganisms and the phagocytosis-dependent signaling pathways (47).
2. The capacity of AuNPs to modulate innate memory induced by microbial agents seems to be specifically restricted to some agents and to some of the memory response parameters (production levels of different cytokines), and appears to be independent of the effect on the primary response. In fact, AuNPs increase the tolerance memory effect induced by MDP on the inflammatory cytokine TNFα, whereas no effect of the primary response to MDP could be observed. In the case of *H. pylori*, while AuNPs could significantly decrease the TNFα primary response, no effect on the tolerance memory response was evident for the same cytokine, while a significant decrease of the IL-10 memory response was observed. Since IL-10 is an anti-inflammatory factor, its decrease would result in an overall increase of inflammation. The circumscribed effects of AuNPs on the memory production of some cytokines in response to some microbial agents could be explained as interference with distinct mechanisms of cell activation and reprogramming, although experimental evidence is currently missing. The physical interaction between AuNPs and microbial agents at priming may lead to a different recognition/activation profile and trigger distinct epigenetic or metabolic pathways responsible of memory establishment.
3. The most striking observation made in this study, which confirms previous reports, is that opposite innate memory responses can be induced by the same agents and in the same conditions in monocytes from different subjects. Thus, the same microbial agent, alone or in combination with AuNPs, can cause potentiation, tolerance or no effect on cells from different donors. This suggests two considerations: first, it is not possible to classify the effects of NPs on innate memory in general terms; second, in order to know whether some NPs (to be used in medical applications) may have a detrimental effect on a patient, it is necessary to obtain a personalized innate memory profile.

## Data Availability Statement

The original contributions presented in the study are included in the article/**Supplementary Material**. Further inquiries can be directed to the corresponding author.

## Author Contributions

VP and FB synthesized and characterised the nanomaterials. BS, SM, TF, AV, and AL contributed to the experimental work. BS and SM planned the study. AD, JH-H, PI, and DB evaluated the results and monitored the experimental work. BS and DB wrote the manuscript. BS prepared the figures and performed the statistical analysis. All authors contributed to the article and approved the submitted version.

## Funding

This work was supported by the EU Commission H2020 projects PANDORA (GA 671881) and ENDONANO (GA 812661), the Italian MIUR InterOmics Flagship projects MEMORAT and MAME, the Italian MIUR/PRIN-20173ZECCM, the Priority program ACBN (Allergy Cancer BioNano Research Centre) of the University of Salzburg, the Cancer Cluster Salzburg, the Research Grant from the University of Salzburg, and the Austrian Science Fund (FWF) Grant Nr. P 29941.

## Conflict of Interest

The authors declare that the research was conducted in the absence of any commercial or financial relationships that could be construed as a potential conflict of interest.

## Publisher’s Note

All claims expressed in this article are solely those of the authors and do not necessarily represent those of their affiliated organizations, or those of the publisher, the editors and the reviewers. Any product that may be evaluated in this article, or claim that may be made by its manufacturer, is not guaranteed or endorsed by the publisher.

## Supplementary Material

Supplementary Figure 1 Effect of AuNPs on the primary response of monocytes to killed *C. albicans*. CD14+ monocytes were stimulated with medium or *C. albicans* (ratio 0.1:1) for 24 h in the presence or absence of AuNPs (20 µg/mL, indicated by blue bars). The inflammatory response is reported in terms of production of IL-6 **(A)**, IL-1Ra **(B)** and MCP-1/CCL2 **(C)** and expressed as ng/106 monocyte. Values from individual donors are depicted concurrent with mean cytokine production. Relevant p values are indicated when < 0.05. n = 4.

Supplementary Figure 2 Effect of AuNPs on the memory response of monocytes primed by *C. albicans*. CD14+ monocytes were exposed to medium or *C. albicans* (ratio 0.1:1) for 24 h in the presence or absence of AuNPs (20 µg/mL, indicated by blue bars), then washed and rested for 6 days. After resting, cells were challenged with LPS (5 ng/mL) for 24 h. Supernatants were collected and cytokine production measured: IL-6 **(A)**, IL-10 **(B)**, IL-1Ra **(C)** MCP-1/CCL2 **(D)**, MIP-1/CCL3 **(E)**, MIP-1b/CCL4 **(F)**, GROa/CXCL1 **(G)**, IP-10/CXCL10 **(H)**, IL-8/CXCL8 **(I)**. Individual donor values are depicted concurrent with mean cytokine production (ng/106 monocytes). n = 4.

Supplementary Figure 3 Effect of AuNPs on the memory response of monocytes primed by *H. pylori*. CD14+ monocytes were exposed to medium or *H. pylori* (at MOI 0.2) for 24 h in the presence or absence of AuNPs (20 µg/mL, indicated by blue bars), then washed and rested for 6 days. After resting, cells were challenged with LPS challenge (5 ng/mL) for 24 h, and supernatant were collected for evaluation of cytokines and chemokines: IL-1a **(A)**, IL-8/CXCL8 **(B)**, MCP-1/CCL2 **(C)**, MIP-1a/CCL3 **(D)**, MIP-1b//CCL4 **(E)**, IP-10/CXCL10 **(F)**. Individual donor values are depicted concurrent with mean cytokine production (ng/106 monocytes). Relevant p values are indicated when < 0.05. n = 4.
