# Supplementary figures and images for: Innate Memory Reprogramming by Gold Nanoparticles Depends on the Microbial Agents That Induce Memory
Source: Front Immunol. 2021 Nov 4;12:751683. doi: 10.3389/fimmu.2021.751683 (PMC8600232; doi:10.3389/fimmu.2021.751683)

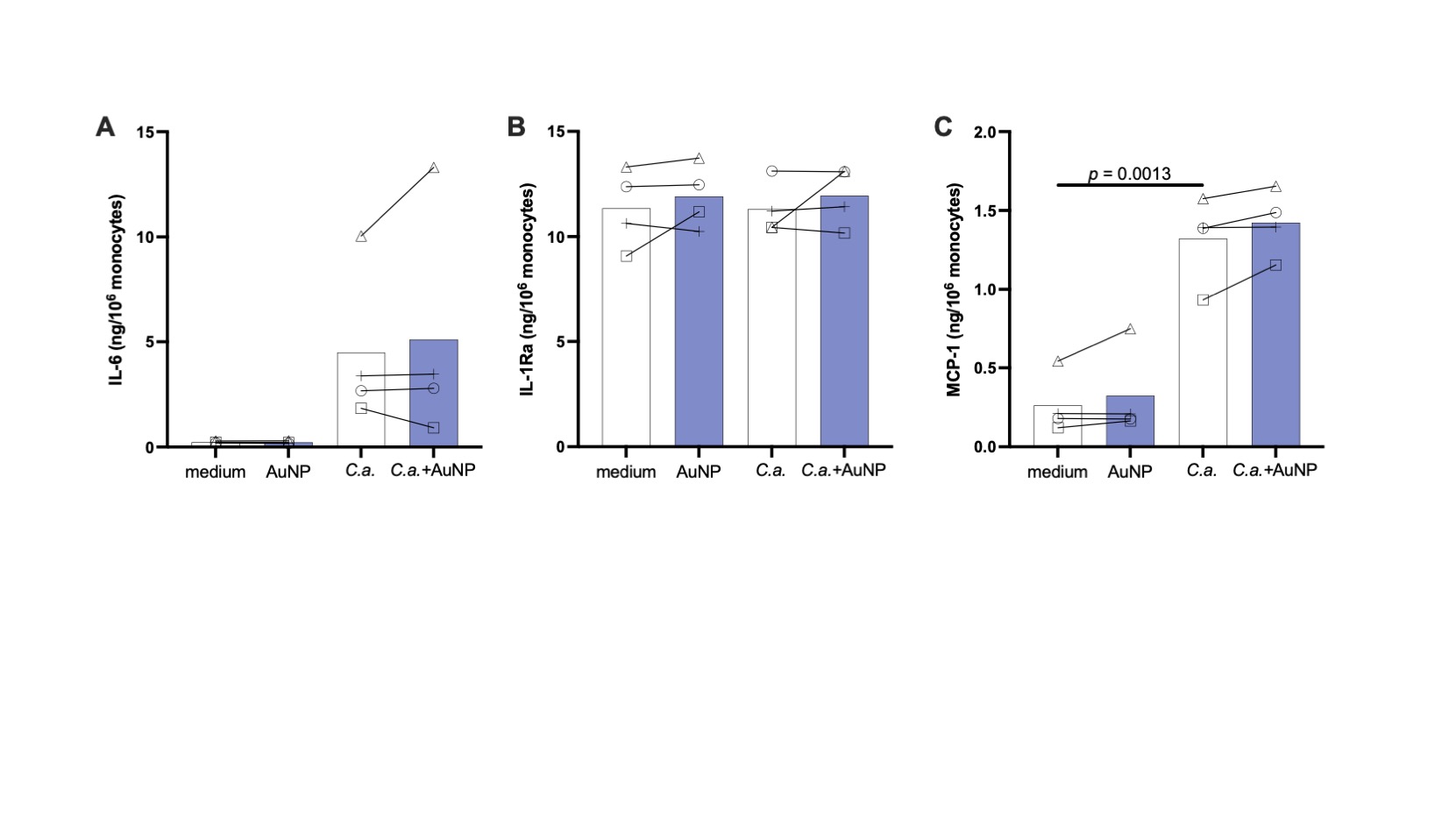

Supplement: Supplementary Figure 1 — Effect of AuNPs on the primary response of monocytes to killed C. albicans. CD14+ monocytes were stimulated with medium or C. albicans (ratio 0.1:1) for 24 h in the presence or absence of AuNPs (20 µg/mL, indicated by blue bars). The inflammatory response is reported in terms of production of IL-6 (A), IL-1Ra (B) and MCP-1/CCL2 (C) and expressed as ng/106 monocyte. Values from individual donors are depicted concurrent with mean cytokine production. Relevant p values are indicated when < 0.05. n = 4. [file Image_1.jpeg]

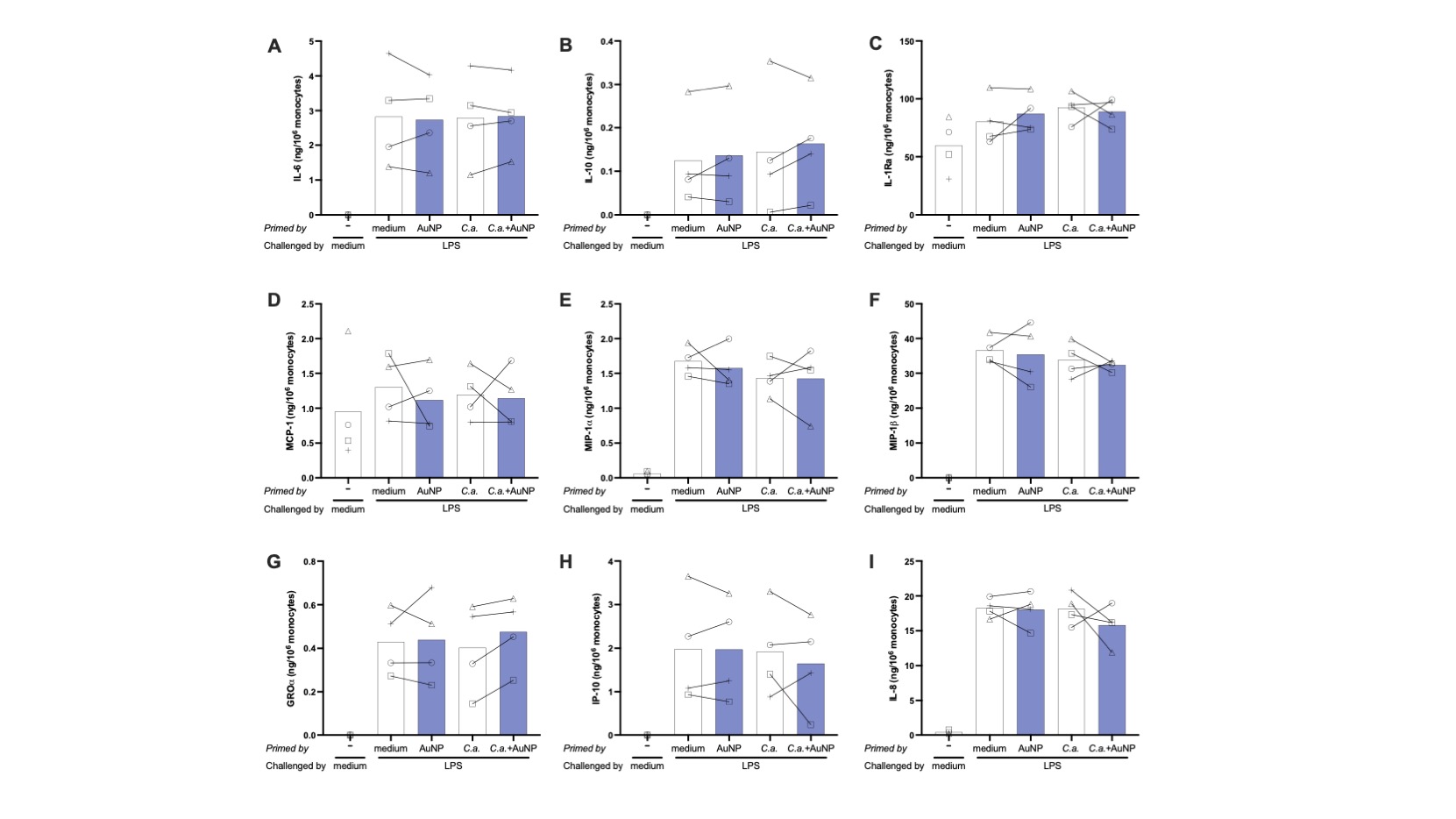

Supplement: Supplementary Figure 2 — Effect of AuNPs on the memory response of monocytes primed by C. albicans. CD14+ monocytes were exposed to medium or C. albicans (ratio 0.1:1) for 24 h in the presence or absence of AuNPs (20 µg/mL, indicated by blue bars), then washed and rested for 6 days. After resting, cells were challenged with LPS (5 ng/mL) for 24 h. Supernatants were collected and cytokine production measured: IL-6 (A), IL-10 (B), IL-1Ra (C) MCP-1/CCL2 (D), MIP-1/CCL3 (E), MIP-1b/CCL4 (F), GROa/CXCL1 (G), IP-10/CXCL10 (H), IL-8/CXCL8 (I). Individual donor values are depicted concurrent with mean cytokine production (ng/106 monocytes). n = 4. [file Image_2.jpeg]

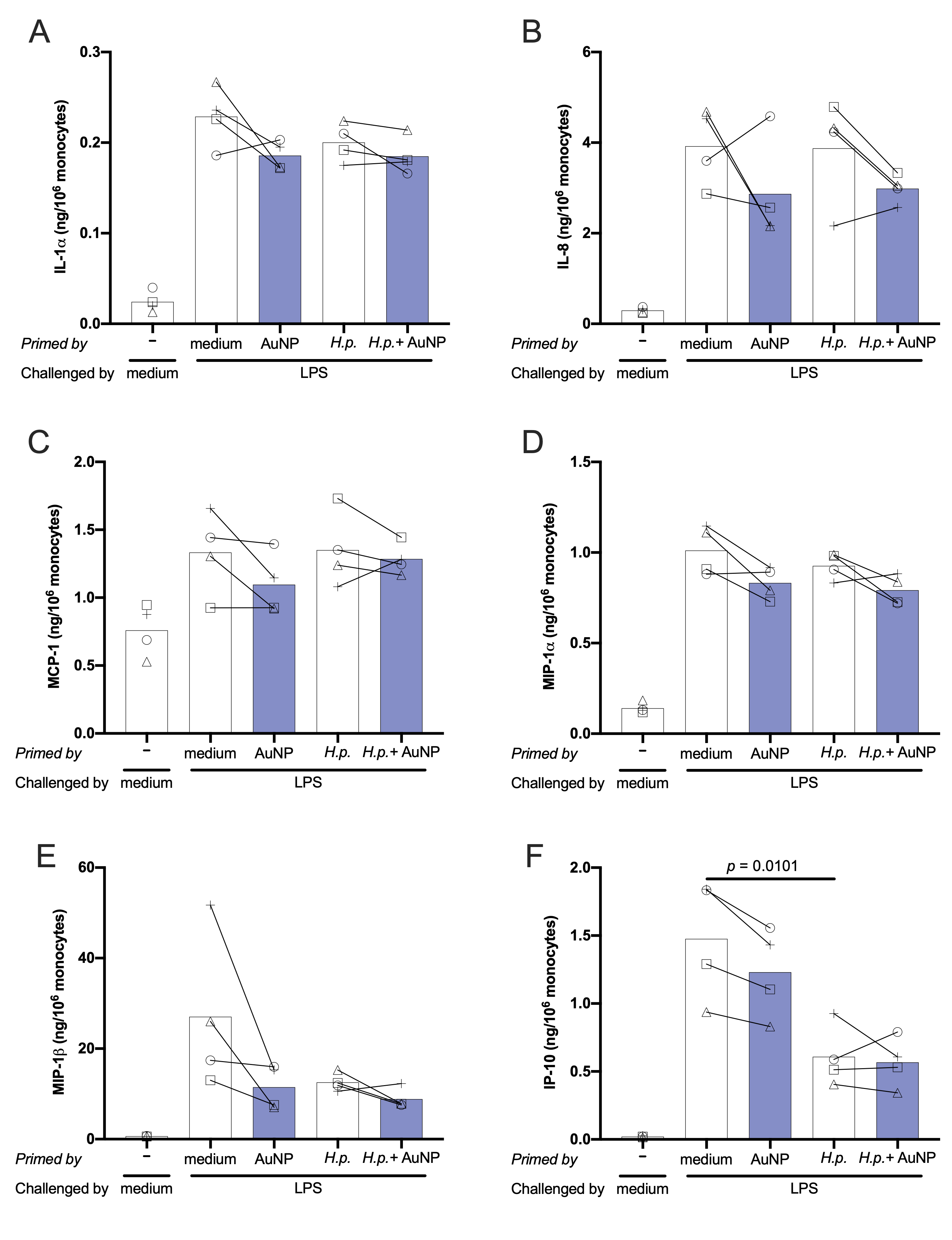

Supplement: Supplementary Figure 3 — Effect of AuNPs on the memory response of monocytes primed by H. pylori. CD14+ monocytes were exposed to medium or H. pylori (at MOI 0.2) for 24 h in the presence or absence of AuNPs (20 µg/mL, indicated by blue bars), then washed and rested for 6 days. After resting, cells were challenged with LPS challenge (5 ng/mL) for 24 h, and supernatant were collected for evaluation of cytokines and chemokines: IL-1a (A), IL-8/CXCL8 (B), MCP-1/CCL2 (C), MIP-1a/CCL3 (D), MIP-1b//CCL4 (E), IP-10/CXCL10 (F). Individual donor values are depicted concurrent with mean cytokine production (ng/106 monocytes). Relevant p values are indicated when < 0.05. n = 4. [file Image_3.jpeg]
